# GDSCalc: A Web-Based Application for Evaluating Discrete Graph Dynamical Systems

**DOI:** 10.1371/journal.pone.0133660

**Published:** 2015-08-11

**Authors:** Sherif H. Elmeligy Abdelhamid, Chris J. Kuhlman, Madhav V. Marathe, Henning S. Mortveit, S. S. Ravi

**Affiliations:** 1 Computer Science Department, Virginia Tech, Blacksburg, Virginia, United States of America; 2 Virginia Bioinformatics Institute, Virginia Tech, Blacksburg, Virginia, United States of America; 3 Computer Science Department, University at Albany—SUNY, Albany, New York, United States of America; University of California, Merced, UNITED STATES

## Abstract

Discrete dynamical systems are used to model various realistic systems in network science, from social unrest in human populations to regulation in biological networks. A common approach is to model the agents of a system as vertices of a graph, and the pairwise interactions between agents as edges. Agents are in one of a finite set of states at each discrete time step and are assigned functions that describe how their states change based on neighborhood relations. Full characterization of state transitions of one system can give insights into fundamental behaviors of other dynamical systems. In this paper, we describe a discrete graph dynamical systems (GDSs) application called GDSCalc for computing and characterizing system dynamics. It is an open access system that is used through a web interface. We provide an overview of GDS theory. This theory is the basis of the web application; i.e., an understanding of GDS provides an understanding of the software features, while abstracting away implementation details. We present a set of illustrative examples to demonstrate its use in education and research. Finally, we compare GDSCalc with other discrete dynamical system software tools. Our perspective is that no single software tool will perform all computations that may be required by all users; tools typically have particular features that are more suitable for some tasks. We situate GDSCalc within this space of software tools.

## Introduction

### Background and Motivation

Civil disobedience [1], addiction [2], emotional behavior [3], social media [4], biology [5], and finance [6] are some of the research topics that are studied using agent-based modeling. Many simulations in these fields represent their populations (of proteins, neurons, institutions, humans) as networks, with vertices and edges denoting agents and their interactions, respectively. One goal of simulation is to understand how information, behaviors, and other contagions propagate through a networked population. There are fundamental aspects of network dynamics common to these and other domains, and other aspects that are domain-specific.

In this work, we present an open access, web-based application called ***GDSCalc*** (or GDSC), which uses a discrete dynamical systems formulation referred to as a **graph dynamical system** (GDS) [7]. (Other names include *finite dynamical systems* and *generalized cellular automata*.)

Informally, a GDS consists of a network; a set of states, an element of which is assigned to each vertex; a function for each vertex that describes how the agent changes its state; and an update procedure that specifies the sequencing of vertex function execution. A GDS computes dynamics by evaluating vertex functions that have dependencies encoded by the (dependency) network, at each time step. A GDS is defined formally below.

The GDSC application can be used for both research and education. As evidence for the former point, we note that three works [8–10] used GDSC to identify *experimentally* dynamical system behaviors that were then rigorously proved as *general* characterizations of GDSs. Thus, GDSC is a useful tool for *experimental mathematics* and *computational mathematics*, where computational studies are used to guide formulation of theorems and provide insights for their proofs. Furthermore, computational results are also useful in their own right; e.g., to explain experimentally-observed behavior of biological systems [5, 11]. Finally, GDSC will be used in a university network science course in Fall 2015.

GDSC works on small to moderate size networks. The reason for this is inherent in the problem of computing the complete dynamics of a GDS: the number of state transitions that must be calculated for an *n*-vertex graph and Boolean (i.e., 2-state) vertex set can be as large as *n*! ⋅ 2^*n*^. For a 100-vertex graph, this is 10^188^ state transitions. Nonetheless, specific reasoning can be done for a much larger class of networks. Furthermore, the theorems alluded to above are constructed for arbitrary numbers of vertices; i.e., the mathematical results are applicable to large (finite) *n*.

GDSs generalize concepts such as cellular automata, Boolean networks, graph automata, and synchronous and sequential discrete dynamical systems. We will return to this topic when we discuss other software systems. GDSs are closely related to a host of other models, such as finite automata, discrete recurrent Hopfield networks, finite state machines, and systolic arrays (see [12] for further references). GDS is a general model of computation which can simulate general and resource-bounded Turing machines [12].

### Contributions

Major contributions of this paper and GDSC follow.

1. **Theoretical and software foundations of GDSCalc capabilities**. We discuss the major elements of GDS theory which provide the basis for the web-application. In particular, the theory is designed to produce a *mental model* [13] (as well as a formal model) for the user, which is then reflected in the user interface (UI), compute engine, and results of GDSC.
2. **Illustrative examples using GDSCalc**. We provide four examples that highlight the usefulness of GDSC for classroom use and research. Although our focus here is on research, our examples will also bring out the usefulness of GDSC for educational purposes. (Terms used here are defined below.) These examples also address features of the system. The first example describes how GDSC was used to find a class of GDS, based on trees (acyclic graphs), that generate particular long-term dynamics: a user-specified limit cycle size. The second example addresses stability and evolution. It illustrates how GDSC was used to find classes of GDS that produce arbitrarily large limit cycles, and limit cycles that form undirected binary hypercubes in attractor graphs. Both of these results significantly extend the state-of-the-art on system stability. A third illustrative use case departs from binary or Boolean vertex state systems to investigate dynamical systems which have any finite number of vertex states. We established what to our knowledge are the first theoretical results of their kind on large state sets, identifying conditions that guarantee that the only long-term dynamics of a system are fixed points, and that these systems can produce bifurcations. These first three sets of results are our own. In a fourth example, we illustrate that GDSC can produce results in the literature [5] from other researchers, demonstrating that GDSC is applicable to research beyond ours. We emphasize that in the first three examples, the experimental results were used to develop intuition and concrete data that enabled us to achieve a primary aim: to rigorously *prove* phenomena about dynamical systems. This justifies our earlier claim that GDSC can be used for experimental and computational mathematics. Furthermore, these examples demonstrate the range of applications that can be investigated with GDSC. Although each set of results can potentially be used in multiple applications, our first through fourth sets of results were motivated by general systems, evolution, social sciences, and biology, respectively. We note that these examples illustrate the human-computer interactive nature of problem solving possible with GDSC. For the first three examples, the problems being addressed can only be defined at a high level (e.g., does a GDS exist that has a requisite set of dynamical properties?). Since there are no known algorithmic solutions to these problems, they require interactive systems [14] that enable a user to test many sets of inputs.
3. **Comparisons of dynamical systems software tools**. We compare GSDC with other dynamical systems tools. We also describe several other dynamical system models which serve as background for the comparisons. GDSC provides unique features not found in other tools. For example, our tool is web-based, meaning that a user need not concern herself with compiling software, third-party libraries, software upgrades, commercial software purchase, system compatibility issues, and high performance resources needed for many of the computations. We also note that GDSC will not perform some analyses available with other software. Our position is that GDSC is a useful tool for some classes of problems, but that other tools are better suited for other problems. An analyst is best served by having access to a collection of tools so that she may select one that is appropriate for a particular task.

The GDSC online environment [15] contains supporting materials including: a PowerPoint presentation for teachers and researchers to introduce/overview the system to users; a user and systems manual; videos that describe how the tool provides immediate usability [16]; and a list of relevant publications.

**Organization**. GDS is formally introduced in the next section, with an example to make the concepts concrete. Research-driven examples are used to demonstrate the utility of the GDSC system; these examples are taken from real research projects using published data. Finally, we itemize features of our system and compare GDSC to other dynamical systems software.

## Analysis

In this section, we formally present the GDS. Then, to make the ideas concrete, we present two vertex functions for vertex state update, followed by examples of GDSs. Variants of the GDS formalism can be found in [17]. In the last subsection, we make a few comments about the GDSC software, relating it to the GDS model.

### Graph Dynamical System Formalism

A GDS is denoted by 𝓢(*X*, *F*, W, *K*). Let *X* denote a directed graph, called a **dependency graph**, with vertex set v[*X*] = {1, 2, …, *n*} and edge set e[*X*]. We use the convention that directed edge (*u*, *v*) means that the state of vertex *u* is used to determine the next state of vertex *v*. To each vertex *v* we assign a state *x*_*v*_ ∈ *K* and refer to this as the **vertex state**; *K* is the **vertex state set**. The 1-neighborhood of a vertex *v* is the set of vertices adjacent to *v* in *X*. Let *n*[*v*] denote the sequence of vertices in the 1-neighborhood of vertex *v* sorted in increasing order such that for each *u* ∈ *n*[*v*], there exists a directed edge (*u*, *v*) ∈ e[*X*]. (If *v* ∈ *n*[*v*], meaning that there is a directed self-loop, then the 1-neighborhood is closed.) In other words, each such *u* is an in-neighbor of *v*, and *d* ^*in*^(*v*) = ∣*n*[*v*]∣, where *d* ^*in*^ is the in-degree of *v*. We write the sequence *x*[*v*] of vertex states corresponding to the vertices in *n*[*v*] as

$$\begin{array}{c} x\left[v\right]=\left(x_{n[v](1)},x_{n[v](2)},\ldots ,x_{n\left[v\right](d^{in}\left(v\right))}\right)\;. \end{array}$$

We refer to *x*[*v*] as the **restricted state**. Here, *n*[*v*](*i*) is the *i*th entry in the sequence. We call *x* = (*x*_1_, *x*_2_, …, *x*_*n*_) the **(system) state**. We denote the (system) state and restricted state at time *t* as *x*(*t*) and *x*(*t*)[*v*], respectively.

The dynamics of changes in vertex states are governed by a sequence $F={\left(f_{v}\right)}_{v=1}^{n}$ of **vertex functions** where each *f*_*v*_:*K* ^*d*^*in*^(*v*)^ → *K* maps as

$$\begin{array}{c} x_{v}\left(t+1\right)=f_{v}(x(t)[v])\;. \end{array}$$

That is, the state of vertex *v* at time *t*+1 is given by *f*_*v*_ evaluated for the restricted state *x*[*v*] at time *t*. To reduce notation, we will often omit the time *t* from the restricted state.

An **update scheme** W governs how the list of vertex functions assemble to a **graph dynamical system map** (see e.g. [7, 18])

$$\begin{array}{c} F:K^{n}⟶K^{n} \end{array}$$

producing the system state at time *t*+1 from that at time *t*; i.e., *x*(*t*+1) = **F**(*x*(*t*)).

We first address the **synchronous** and **sequential** update schemes. In the former case we have the synchronous (parallel) GDS map

$$\begin{array}{c} F\left(x_{1},x_{2},\ldots ,x_{n}\right)=\left(f_{1}\left(x\left[1\right]\right),f_{2}\left(x\left[2\right]\right),\ldots ,f_{n}\left(x\left[n\right]\right)\right)\;. \end{array}$$

We refer to this subclass of GDS as **synchronous dynamics systems** (SyDS), since all vertex functions are executed simultaneously (i.e., in parallel); it is sometimes referred to as **generalized cellular automata**. In the latter case we consider permutation update sequences. We first introduce the notion of ***X*-local functions**. Here, the *X*-local function *F*_*v*_:*K* ^*n*^ → *K* ^*n*^ is given by

$$\begin{array}{c} F_{v}\left(x_{1},\ldots ,x_{n}\right)=\left(x_{1},\ldots ,x_{v-1},f_{v}\left(x\left[v\right]\right),x_{v+1},\ldots ,x_{n}\right)\;; \end{array}$$

i.e., *F*_*v*_ updates only the *v*th component of the system state. Using *π* = (*π*_1_, …, *π*_*n*_) ∈ *S*_*X*_ (the set of all permutations of v[*X*]) as an update sequence, the corresponding asynchronous (or sequential) GDS map **F**_*π*_:*K* ^*n*^ → *K* ^*n*^ is given by

$$\begin{array}{c} F_{\pi }=F_{\pi_{n}}\circ F_{\pi_{n-1}}\circ \cdots \circ F_{\pi_{2}}\circ F_{\pi_{1}}\;, \end{array}$$

which is the composition of the *X*-local functions. We refer to this class of asynchronous systems as **(permutation) sequential dynamical systems** (SDS).

A generalization of the two previous update schemes is **block sequential**. In this scheme, the vertices are partitioned into a sequence of *q* sets or blocks *B* = (*B*_1_, *B*_2_, …, *B*_*q*_). The vertex functions for the vertices in each block are executed simultaneously, with sequential ordering between consecutive blocks. Let *π*_*B*_ = (*π*_*B*_1__, …, *π*_*B*_*q*__) be a block permutation. We have the *X*-local function, for *l* ∈ {1, …, *q*}, *F*_*π*_*B*_*l*___:*K* ^*n*^ → *K* ^*n*^, where the *i*th entry in *F*_*π*_*B*_*l*___ is the identity map if vertex *i* ∉ *B*_*l*_ and is *f*_*i*_ if vertex *i* ∈ *B*_*l*_. We have the block sequential GDS map

$$\begin{array}{c} F_{\pi_{B}}=F_{\pi_{B_{q}}}\circ F_{\pi_{B_{q-1}}}\circ \cdots \circ F_{\pi_{B_{2}}}\circ F_{\pi_{B_{1}}}\;, \end{array}$$

and refer to it as a **block sequential dynamical system** (BSDS). When the size of each block is one, a block sequential GDS map reduces to a sequential GDS map, and when all vertices are in one block, the block sequential map reduces to a synchronous GDS map. We describe all three types of maps here because different works in the literature may use only one of the update methods.

To this point, we have described **fair word orders**; that is, each vertex appears exactly once in a (block) permutation. **Unfair word orders** [18], where vertices may appear more than once in a (block) permutation, are also studied. If unspecified, the convention is to assume a fair word order.

The **phase space** Γ(**F**) of the GDS map **F** is a directed graph with vertex set *K* ^*n*^ and edge set $\left\{(x,\text{F}(x))|x\in K^{n}\right\}$. A state *x* for which there exists a positive integer *p* such that **F** ^*p*^(*x*) = *x* is a **periodic point**, and the smallest such integer *p* is the **period** of *x*. If *p* = 1 we call *x* a **fixed point** of **F**. A state that is not periodic is a **transient state**. Classically, the **omega-limit set** of *x*, denoted by *ω*(*x*), is the set of accumulation points of the sequence {**F** ^*k*^(*x*)}_*k* ≥ 0_. In the finite case, the omega-limit set (also called a **(limit) cycle**, **(periodic) orbit**, **limit set**, or **attractor**) is the unique periodic orbit reached from *x* under **F**.

Given two update sequences *π* and *π*′, if **F**_*π*_ and **F**_*π*′_ give the exact same state transitions; i.e., **F**_*π*_(*x*) = **F**_*π*′_(*x*) for every *x* ∈ *K* ^*n*^, then the GDS maps are equal; i.e., **F**_*π*_ = **F**_*π*′_, and we say that the maps are **functionally equivalent**. If the limit cycle structures for the two maps are the same, to within an isomorphism, then we say the two maps are **cycle equivalent** [7]. Cycle equivalence describes long-term dynamics. If two maps are functionally equivalent, then they are cycle equivalent. Functional and cycle equivalence can be computed for any pair of GDSs, irrespective of update sequence.

### Example Vertex Functions

First, we introduce threshold and bithreshold vertex functions. We confine ourselves to Boolean systems so that *K* = {0,1}. We write *d* ^*in*^ for *d* ^*in*^(*v*) and assume that *v* ∈ *n*[*v*]. A Boolean **threshold function** *θ*_*v*, *k*, *d*^*in*^_ : *K* ^*d*^*in*^^ → *K* is defined by

$$\begin{array}{c} \theta_{v,k,d^{in}}\left(x_{1},\ldots ,x_{d^{in}}\right)=\{\begin{array}{cc} 1, & \text{if}\;\sigma_{v}\left(x_{1},\ldots ,x_{d^{in}}\right)\geq k\;\text{and}\\ 0, & \text{otherwise,} \end{array} \end{array}$$ (1)

where $\sigma_{v}(x_{1},\ldots ,x_{d^{in}})=|\{1\leq j\leq d^{in}|x_{j}=1\}|$.

Threshold functions are used in modeling biological systems [5, 19], and social behaviors (e.g., joining a revolt, technology adoption, spread of rumors, and other social contagions), see, e.g., [20–23]. A **bi-threshold function** is a function *θ*_*v*, *k*_01_, *k*_10_, *d*^*in*^_ : *K* ^*d*^*in*^^ → *K* defined by

$$\begin{array}{c} \theta_{v,k_{01},k_{10},d^{in}}\left(x_{1},\ldots ,x_{d^{in}}\right)=\{\begin{array}{cc} \theta_{v,k_{01},d^{in}}, & \text{if}\;x_{v}=0,\\ \theta_{v,k_{10},d^{in}}, & \text{if}\;x_{v}=1\;. \end{array} \end{array}$$ (2)

We call *k*_01_ the **up-threshold** and *k*_10_ the **down-threshold**. The up-threshold *k*_01_ denotes the minimum number of vertices in *n*[*v*] that are required to be in state 1 in order for *v* to transition to 1 when its state is 0. When *x*_*v*_ = 1, if the number of vertices in *n*[*v*] that are in state 1 (including *v*, in a closed neighborhood) is at most *k*_10_−1, then *v* transitions to 0. Otherwise *x*_*v*_ does not change. Alternatively, using *σ*_*v*_, we have the following equivalent description. If we let *σ*_*v*_ = *σ*_*v*_(*x*_1_, …, *x*_*d*^*in*^_) for vertex *v*, then *v* transitions from state 0 to state 1 if *σ*_*v*_ ≥ *k*_01_. A vertex *v* transitions from 1 to 0 if *σ*_*v*_ < *k*_10_. Otherwise, *x*_*v*_ remains unchanged.

When *k*_01_ = *k*_10_, the bi-threshold function behaves like a standard threshold function.

These two thresholds are integers (without loss of generality), and the effective ranges of the thresholds are *k*_01_ ∈ [0, *d* ^*in*^+1] and *k*_10_ ∈ [1, *d* ^*in*^+2]. When *k*_01_ = 0 for *v*, the vertex will transition from state 0 to 1, irrespective of the states of its neighbors. When *k*_01_ = *d* ^*in*^+1, *v* will remain in state 0 irrespective of the states of its neighbors. However, from a practical standpoint, we allow *k*_01_ ∈ ℕ because this enables thresholds to be more easily specified. For example, if we have a collection of vertices *V*_*c*_ whose states should remain 0, then it is easier to set *k*_01_ = *n* for all *v* ∈ *V*_*c*_ (since *x*_*v*_ = 0, *v* cannot have *n* vertices in its closed neighborhood that are in state 1). This value of *k*_01_ = *n* applies to all vertices in *V*_*c*_ without inspecting their degrees. In an analogous manner, *k*_10_ = *d* ^*in*^+2 assigned to *v* ensures that it always transitions down, from 1 to 0. The limiting case occurs when *v* ∉ *n*[*v*], because in this case, *v* and all of its *d* ^*in*^ neighbors could be in state 1. We have that the number of vertices in state 1 is *d* ^*in*^+1 < *k*_10_ = *d* ^*in*^+2, which ensures the down-transition to 0. Similarly, when *x*_*v*_ = 1, if we set *k*_10_ = 1, then *v* will never transition down because it will never be true that the number of vertices in state 1 in the closed neighborhood of *v* is < *k*_10_.

A second vertex function is the nor function for a Boolean system; nor: *K* ^*d*^*in*^^ → *K*, defined by

$$\begin{array}{c} \text{nor}\left(x_{1},\ldots ,x_{d^{in}}\right)=\Pi_{j=1}^{d^{in}}\left(1+x_{j}\right) \end{array}$$ (3)

where all (1+*x*_*j*_) are modulo 2. Hence, the only way for a nor vertex function to evaluate to 1 is for all inputs to have state 0. GDSs that utilize nor functions are studied because they have interesting properties, such as limit cycles in phase spaces are not fixed points [18].

### GDS: Illustrative Examples

**Fig 1:**
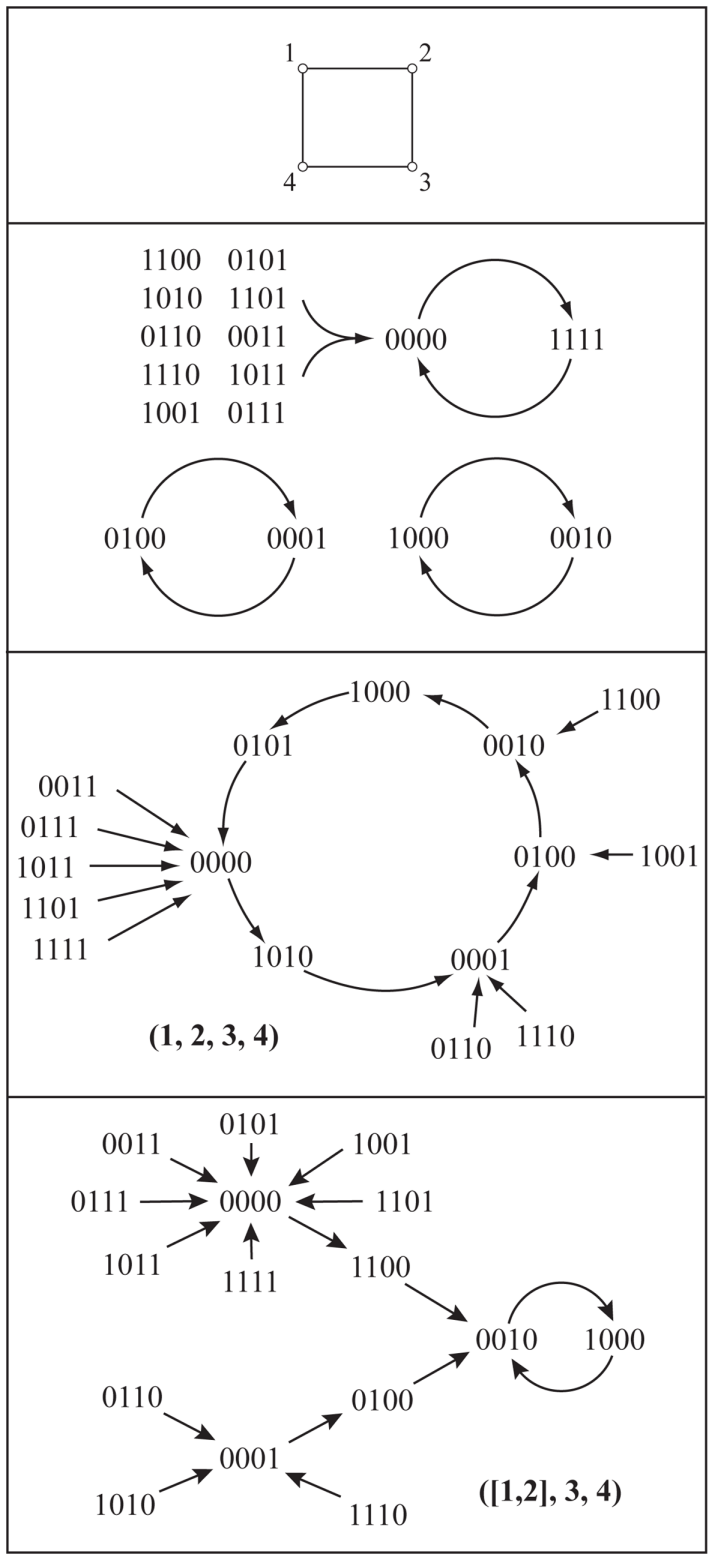
Phase spaces for three GDSs. The bidirected graph *X* = Circle_4_ (top), and the phase spaces of the synchronous GDS; a sequential GDS with update permutation *π* = (1,2,3,4); and a block sequential GDS with block permutation *π*_*B*_ = ([1, 2],3,4). All vertices use the nor function.

We provide phase spaces for GDS maps wherein the dependency graph *X* is a bidirected Circle_4_ graph on four vertices, the vertex state set *K* = {0,1}, and all vertex functions are nor functions. We compare the phase spaces of the synchronous GDS, and particular sequential and block sequential GDSs. Fig 1 provides the graph and three phase spaces. The top phase space is for the synchronous GDS; the middle phase space is for a sequential GDS where *π* = (1,2,3,4); and the lower phase space is for a block sequential GDS with *π*_*B*_ = ([1, 2],3,4). The block sequential permutation has blocks *B*_1_ = {1,2}, *B*_2_ = {3}, and *B*_3_ = {4}, meaning that *f*_1_ and *f*_2_ execute in parallel, followed by *f*_3_, and then *f*_4_, and hence is close to the sequential permutation.

Given the state (0,0,0,0), the next state is **F**(0,0,0,0) = (1,1,1,1) for synchronous update, **F**_*π*_(0,0,0,0) = (1,0,1,0) for sequential update (with the particular permutation), and **F**_*π*_*B*__(0,0,0,0) = (1,1,0,0) for the specified block permutation. Hence, the next states are different for the three GDSs.

Overall, it is apparent that the three phase spaces are different. The long-term dynamics are also different, as described by the limit cycles. The sequential GDS has one 7-cycle; the synchronous GDS contains 2-cycles with multiplicity 3 (i.e., there are three 2-cycles); and the block sequential GDS has one 2-cycle (i.e., multiplicity 1).

In each GDS, state (0,0,1,1) is a transient state (it is not an element of a limit cycle). In the sequential and synchronous systems, it is part of a transient of length 1; i.e., there is one transition from state (0,0,1,1) before the system reaches a state on a limit cycle. This is the maximum transient length for these two GDSs. In contrast, for block sequential update, state (0,0,1,1) is part of a transient of length 3. In this system, this is the maximum transient length, and there are 10 transients of length 3.

A **basin of attraction**, as used here, is the set of all states that (eventually) transitions to, or is contained in, an attractor. For example, in the synchronous GDS, the two states (0,1,0,0) and (0,0,0,1) form a 2-cycle attractor, and since there are no transient states that (eventually) transition to these two states, these two states form the basin of attraction for this attractor. There are three basins of attraction for the synchronous GDS. For each of the sequential GDS and the block sequential GDS, there is one attractor and one basin of attraction; this means that all states eventually transition to the respective limit cycle.

No pair of these three GDSs are functionally equivalent because they do not produce the same state transitions; i.e., the same phase spaces. Based on the cycle lengths and multiplicities given above, no two GDSs are cycle equivalent. See Table A in S1 File for GDSC analyses and data described in this example.

### Overview of GDSC Analysis

**Fig 2:**
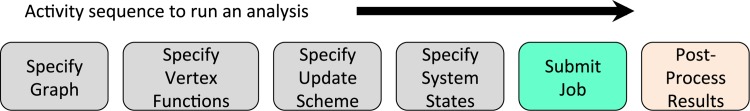
Sequence of high-level user activities to run an analysis in GDSC.

Fig 2 provides a simple activity diagram for specifying an analysis with GDSC. These activities reflect the theoretical model presented above. The user specifies a graph *X*. Thirteen graph templates are available for composing graphs, which include lattices, cliques, bicliques, and trees. A vertex function *f*_*v*_ is assigned to each vertex *v*. Currently, 15 types of vertex functions exist in the library from which a user can choose. The threshold, bithreshold, and nor functions of Eqs (1), (2), and (3) are three such types. Since many of these functions take user-specified inputs, the range of functions that can be evaluated is significantly greater than 15. An update scheme is then chosen. Any of the schemes described above can be selected, including fair and unfair word orders. As the fourth step, evaluation of all system states is the typical choice. After a job is submitted, its status can be monitored. Upon completion, results in the form of an XML file that contain all system state transitions, all functionally equivalent GDS maps, and all cyclic equivalent GDS maps can be viewed. Plots are also produced from the XML file.

## Results

We provide four example research problems solved with GDSC. The first three illustrate our use of GDSC to compute dynamics, which we then used to prove more general results. These theoretical results have been published [8–10]. The fourth study demonstrates the wider applicability of GDSC by illustrating how dynamical systems used by other researchers (e.g., [5]) can also be modeled in this framework. Experimentally, we can evaluate 20-vertex graphs. This limitation is due to memory consumption; we are working to increase this limit. Also, at least for the first three examples below, it is very difficult to envision that the theoretical results could have been produced without a tool like GDSC to perform computations that, in turn, guide or inform the theoretical studies.

Before moving to these examples, we note that the power of GDSC grows with user expertise in dynamical systems (although this expertise is not required). As one example, it was proved in [7] that for any network *X* that is a tree, all sequential update permutations produce the same cycle equivalence class. Consequently, naively computing phase spaces (and limit cycles) for all permutations of a 20-vertex tree requires determining 20! ⋅ 2^20^ ≈ 10^24^ state transitions. But using the above result, this number can be reduced by a factor of 20! ≈ 10^18^ because now only one of the 20! permutations need be evaluated. Other examples of theoretical results that can be used to reduce computational load can be found in, for example, [5, 7, 8, 24, 25].

### Tree Structures and Limit Cycle Sizes for Sequential and Synchronous Update

In this work, we investigated the sizes of limit cycles that can be generated for sequential and synchronous update schemes of bithreshold GDS maps, where vertex functions are described by Eq (2). We proved significant differences for these schemes for arbitrary graphs. We showed that bithreshold synchronous GDS maps can have limit cycles of length at most 2 [8]. We also showed for bithreshold systems that if Δ = *k*_10_ − *k*_01_ ≤ 1, then sequential GDS could only produce fixed points. This motivated the question of how large limit cycles could be in sequential update systems if the condition on Δ is violated. To this end, we used GDSC to experimentally explore a range of graph classes and instances to identify sequential GDSs that produce arbitrarily long limit cycles, for the thresholds (*k*_01_, *k*_10_) = (1,3), which minimally violate the condition on Δ.

By “minimally violated,” we mean conditions that just make a condition false. For example, if *k*_10_ = 3 and *k*_01_ = 1, then Δ = 2, which minimally violates the condition Δ ≤ 1. Δ = 3 also violates the condition, but not minimally. Minimally violating a condition is useful to investigate because we determine whether such violations lead to large differences in phase space properties, such as the length of the longest limit cycle.

**Fig 3:**
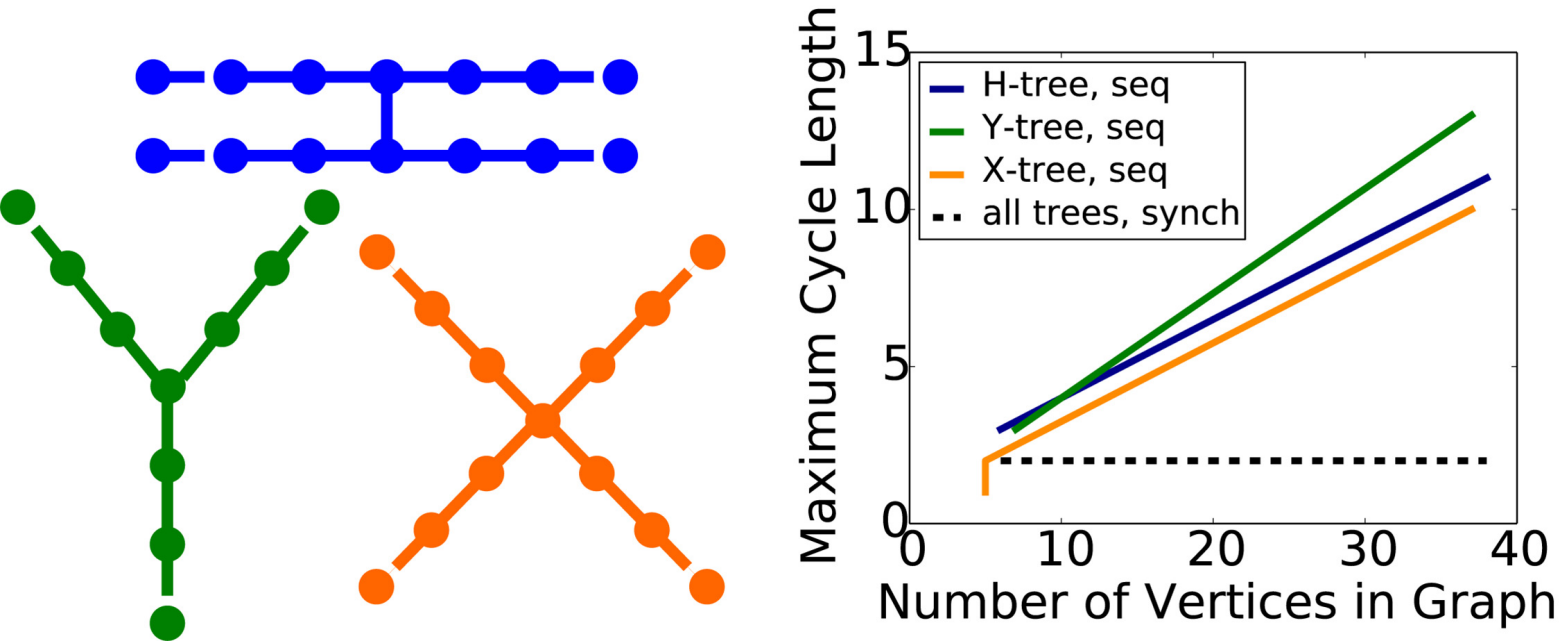
Experimental results generated with GDSC showing the maximum cycle length versus number of graph vertices when the condition Δ = *k*_10_ − *k*_01_ ≤ 1 is minimally violated. The goal is to produce GDSs that generate larger limit cycles than fixed points. The three tree structures on the left were found experimentally, and results were used to prove that there exist graphs with particular structures that produce limit cycles of any specified size for sequential update (see plot at right). Functional forms of these relationships are given in [8]. These structures differ in the smallest limit cycles they can produce and in the maximum cycle size for a given number of vertices. Data for the sequential update scheme are presented as solid lines; limit cycle size increases without bound for increasing graph size. Data for synchronous update, for all graph structures, is the dashed black curve.

Three such graph classes are shown in Fig 3 at the left (so-called *H*, *Y*, and *X* trees, based on their structures). The plot on the right shows the maximum cycle length as a function of number of graph vertices when the condition on Δ ≤ 1 is minimally violated with *k*_01_ = 1 and *k*_10_ = 3, so that Δ = 2. Given *H* trees as a starting point, *Y* trees, for sufficiently large *n*, produce larger limit cycles for the same number of vertices. Also, unlike the other two structures, *X* trees can produce fixed point (beyond the obvious case of the zero state) and 2-cycle limit sets. The issue of designing (dynamical) systems to produce particular behaviors is of general interest (e.g., [26]).

The dashed curve in Fig 3 represents data for synchronous update. As noted above, synchronous systems can have cycle lengths of at most 2. Thus, this figure also illustrates the impact of update scheme on limit cycle size: the size difference between synchronous and sequential update schemes can be arbitrarily large. Tables B through D in S1 File contain GDSC analyses for *X*, *Y*, and *H* trees of this section.

### Multi-State, Multi-Threshold Systems

**Fig 4:**
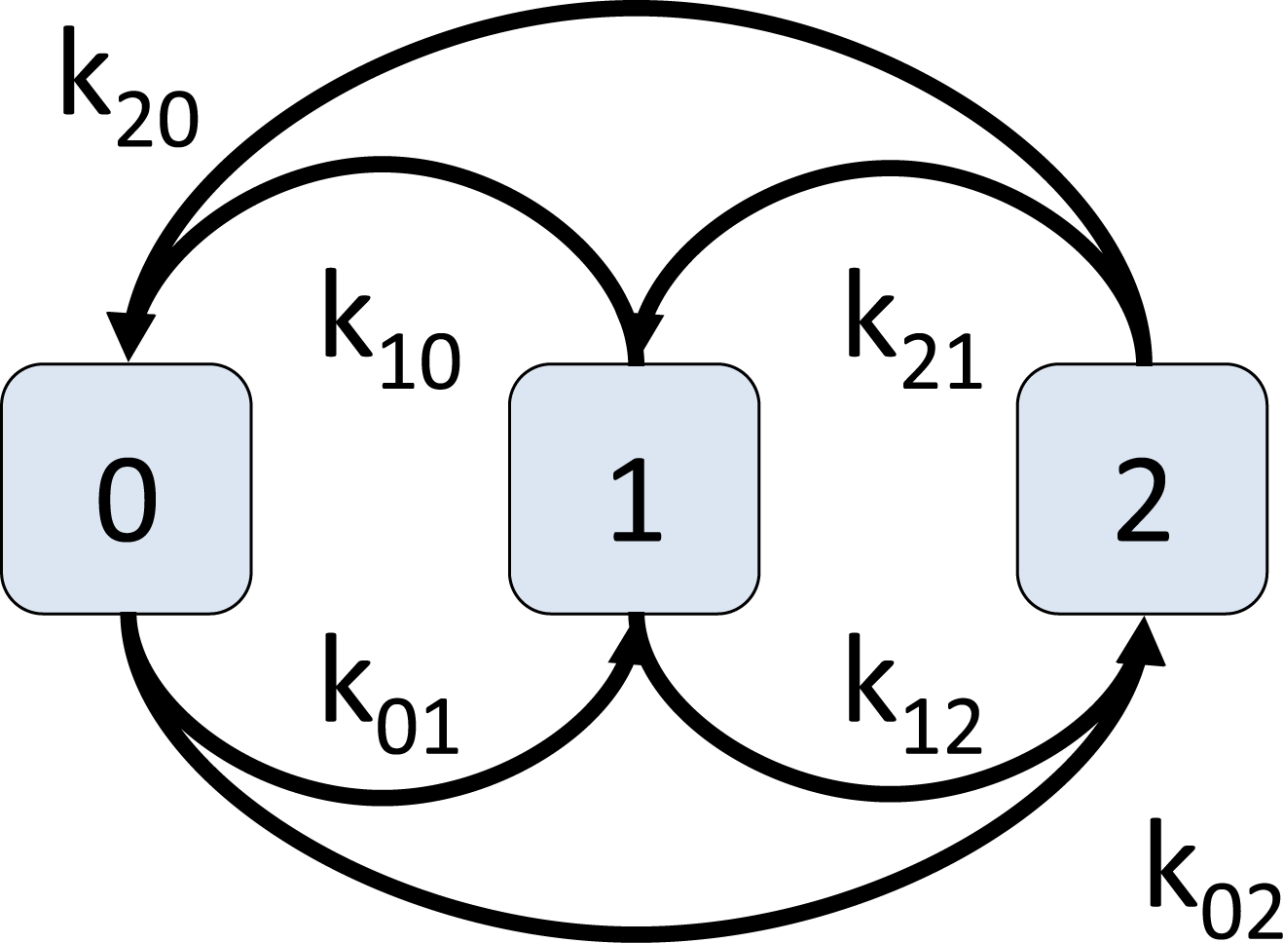
A 3-vertex-state, multi-threshold system where each vertex state transition is governed by a distinct threshold. Threshold *k*_*ij*_ governs the transition from state *i* to *j*.

Much of the work on discrete dynamical systems focuses on Boolean systems (with *K* = {0,1}) where state 0 represents an inactive or non-participating state and state 1 represents an active or participating state. From a social dynamics perspective, there is considerable motivation for investigating systems with more states (e.g., [27]) so that a finer resolution of behavior can be assessed. Macy [21], in studying social behavior, cites Elster [28]: “although the assumption of a dichotomous independent variable—the decision [by humans] to cooperate—is convenient for many purposes, it is often unrealistic. Often, the problem facing the actor is not *whether* to contribute, but *how much* to contribute.” In [10], we study GDSs where there can be any finite number *r* of states, that is, *K* = {0, 1, 2, …, *r* − 1}. We explore sequential update behavior to find necessary conditions for these GDSs to produce only fixed points as limit sets. The vertex state transitions for the case *r* = 3 are given in Fig 4. The threshold *k*_*ij*_, for *i* < *j*, is the minimum sum of the states of the vertices in *n*[*v*] that will cause *v* to transition from state *i* to *j*. For threshold *k*_*ij*_ with *i* > *j*, a vertex *v* transition from state *i* to *j* if the sum of the states of the vertices in *n*[*v*] is strictly less than *k*_*ij*_. We note that (biological) regulatory networks [29, 30] use multi-state vertex states to describe, for example, multiple expression levels. Selected main results for sequential update follow.

First, we show the theoretical result, for the *r* = 3 case, that there are four inequalities involving the six thresholds (Fig 4) such that if all conditions are satisfied, then a sequential GDS will produce only fixed points as limit sets. These *fixed point conditions*, specified in terms of only thresholds and constants, are [10]:

(i) Δ_01_ ≤ min{−*C*_1_ − 1, *C*_1_ + 3},
(ii) Δ_12_ ≤ min{*C*_1_ − *C*_2_ − 3, −*C*_1_ + *C*_2_ + 5},
(iii) (*k*_21_ + *k*_12_) + (*k*_10_ + *k*_01_) − 4*k*_02_ ≤ −*C*_2_ − 1 and
(iv) −(*k*_21_ + *k*_12_) − (*k*_10_ + *k*_01_) + 4*k*_20_ ≤ *C*_2_ + 11,

where Δ_01_ = *k*_10_ − *k*_01_, Δ_12_ = *k*_21_ − *k*_12_, and *C*_1_ and *C*_2_ are constants. Note that in some sense, there is flexibility in assigning *C*_1_ and *C*_2_, but specifying them to create a broader range of permissible thresholds for one inequality can restrict the range of thresholds in another. The numbers of inequalities, thresholds, and constants grow as *r* increases.

**Fig 5:**
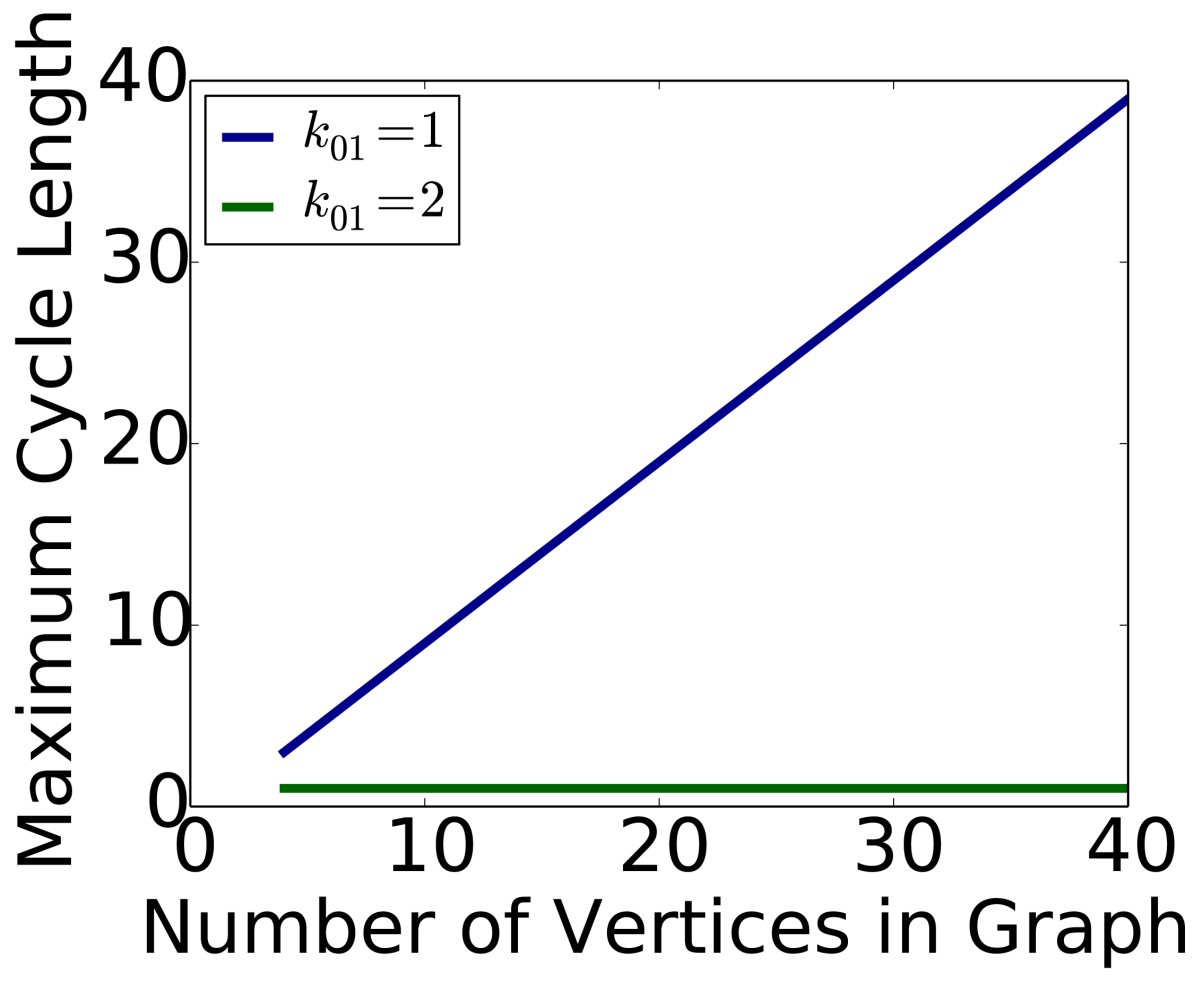
Results that guided proofs of bifurcations in *r*-state sequential GDS maps. When the threshold vector is *k* = (*k*_01_, *k*_10_, *k*_12_, *k*_21_, *k*_02_, *k*_20_) = (2,3,6,6,4,5), the maximum cycle size ℓ = 1 is fixed for all *n* of Circle_*n*_ because the fixed point conditions do not depend on *n*. When *k*_01_ is reduced from 2 to 1, the maximum cycle length increases with number of vertices in Circle_*n*_ graphs as ℓ = *n* − 1.

Second, the bounds on these conditions are sharp, which we demonstrate through computations. That is, there exist GDS maps such that if any one of the conditions above is minimally violated, then the GDS can produce arbitrarily large limit sets. For example, consider a Circle_*n*_ graph, *n* ≥ 4 (a Circle_4_ graph is shown in Fig 1), with the state transition diagram in Fig 4. We use the permutation *π* = (1, 2, …, *n*) for the sequential GDS. We take *C*_1_ = −2 and *C*_2_ = −6 because these maximize the right hand sides of conditions (*i*) and (*ii*). The conditions become (*i*) Δ_01_ ≤ 1 and (*ii*) Δ_12_ ≤ 1. Consider the multi-threshold vector *k* = (*k*_01_, *k*_10_, *k*_12_, *k*_21_, *k*_02_, *k*_20_) = (2,3,6,6,4,5). It can be shown that these thresholds satisfy the four inequalities above; therefore, the GDS will produce only fixed points as limit sets. Thus, in Fig 5, the green curve, corresponding to *k*_01_ = 2, is flat: the maximum cycle length is 1 (i.e., fixed points) irrespective of *n*. Now, consider an identical system except change *k*_01_ from 2 to 1. This minimally violates condition (*i*): Δ_01_ = *k*_10_ − *k*_01_ = 3 − 1 = 2, which is not ≤ 1. It can be shown that the other three conditions are still satisfied. The largest limit cycle in this latter system, where *k*_01_ = 1, is shown in Fig 5. We see that the maximum limit cycle length now grows with *n*. For example, for an *n* = 40 vertex Circle graph, the maximum cycle length is 39. In general, the maximum cycle length, for this particular GDS, is *n* − 1. Thus, we see that by taking a *n*-vertex graph, and using two GDSs, one with *k*_01_ = 2 and one with *k*_01_ = 1, but otherwise are the same, the difference in the maximum limit cycle lengths for the two systems is (*n* − 1)−1 = *n* − 2. Hence, this result demonstrates that minimal violation of a single fixed point condition produces a *bifurcation*: GDSs that transition from generating only the smallest limit sets to arbitrarily large ones. Table E in S1 File contains GDSC analyses and data for conditions in Fig 5.

### Stability and Biological Evolution

Researchers have long used Boolean networks (essentially, GDSs or automata with *K* = {0,1}) to study biological systems [11]. One issue that bears on evolution and species fitness is stability. The question is: how stable are genetic structures? One approach to answer this question is to start by computing the phase space of a GDS. Then, one vertex state of one (system) state in a limit cycle is flipped (from 0 → 1 or vice versa) and one determines whether the long-term dynamics end up in the same limit cycle or a different one. One forms an **attractor graph**, where each vertex represents a limit set (or attractor), and a directed edge from attractor *A*_*i*_ to attractor *A*_*j*_ means that one vertex state of a system state in *A*_*i*_ can be flipped such that the resulting state lays in the basin of attraction of (i.e., transitions to a state in) *A*_*j*_.

Essentially, the more limit cycles *in the attractor graph*, referred to as **ergodic sets** or strongly connected components of the graph, the more biologically diverse the GDS. Most work (e.g., [19, 31]) on Boolean networks demonstrates that limit cycles in attractor graphs are (*i*) very few in number, and (*ii*) almost always fixed points, and occasionally 2-cycles. These results suggest that mutations all lead to the same one or two biological states.

**Fig 6:**
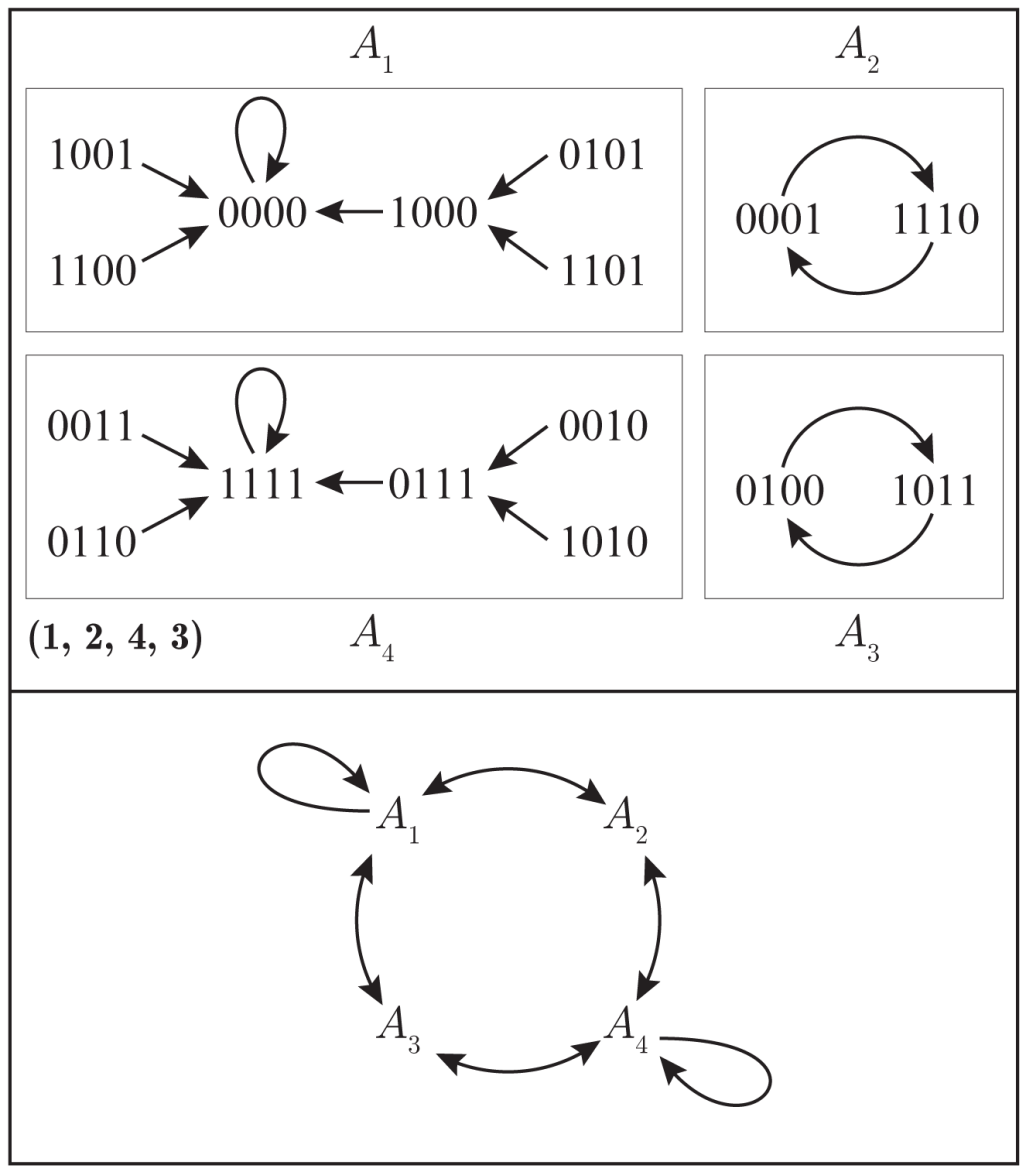
Phase space for a bithreshold sequential GDS and an ergodic set for the attractor graph. A bithreshold sequential GDS with (*k*_01_, *k*_10_) = (1,3) and permutation *π* = (1,2,4,3). The four basins of attraction in the phase space are highlighted (top). The resulting attractor graph (bottom) forms an ergodic set (i.e., a strongly connected component) of size 4.

As an illustrative example, we consider a sequential GDS map **F**_*π*_ with bithreshold vertex functions and (*k*_01_, *k*_10_) = (1,3) on the graph Circle_4_. We use sequential update with permutation *π* = (1,2,4,3). In the top of Fig 6, we show the phase space, and highlight the four attractor basins *A*_1_ through *A*_4_. We slightly abuse notation by letting *A*_*i*_, 1 ≤ *i* ≤ 4, represent an attractor and attractor basin. We draw the four attractors as vertices in the lower portion of the figure. To join these vertices in the attractor graph, we evaluate edges *A*_*i*_ → *A*_*j*_ as described immediately above. We see in attractor *A*_1_, which consists only of the fixed point state (0,0,0,0), that if we flip the vertex state *x*_4_ from 0 to 1, then the state (0,0,0,1) is in the basin of attractor *A*_2_, and hence we have directed edge (*A*_1_, *A*_2_). As another example, if we take state (1,0,1,1) on the limit cycle in *A*_3_, and change the state of *x*_4_ from 1 to 0, then the state (1,0,1,0) is in the basin of attraction of *A*_4_, yielding the directed edge (*A*_3_, *A*_4_). With similar arguments, we arrive at the attractor graph at the bottom of Fig 6, which forms one 4-cycle. A noteworthy point is that the directed edges between pairs of attractors are bi-directed in this example. They need not be; they only need to form a strongly connected component (i.e., each vertex *A*_*j*_ needs to be reachable from each *A*_*i*_). Thus, *A*_1_, *A*_2_, *A*_3_, and *A*_4_ form an ergodic set of size 4. Table F in S1 File contains the information for this analysis in GDSC. We now describe the use of GDSC to experimentally identify Boolean bithreshold and threshold GDSs that are rich in attractor structure.

We searched experimentally using GDSC for graphs *X* in GDSs that generate many attractor graph limit cycles, and cycle structures larger than fixed points. The analyses we use here are identified in Table G in S1 File. We found that graphs composed of cliques of at least five vertices (i.e., *K*_5_) in bithreshold GDSs (see Eq (2)) with (*k*_01_, *k*_10_) = (*q*_*i*_ − 1, *q*_*i*_ − 1), where *q*_*i*_ is the number of vertices in the *i*th clique, can produce both features. Finding these GDSs took considerable experimental work. Then the issue of how to connect multiple cliques together needed to be resolved, and we used experiments again for this purpose. We built up intuition for permissible connectivity patterns among cliques in order to generate formal proofs of behaviors; cliques cannot be connected in any arbitrary fashion to achieve our purposes.

**Fig 7:**
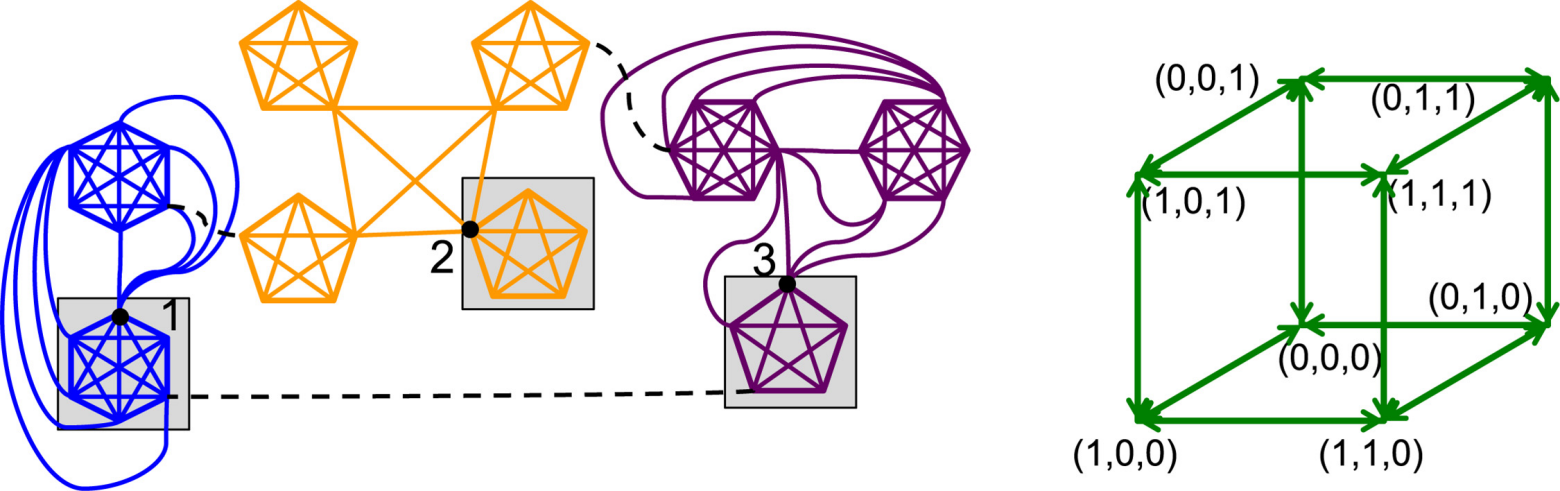
Representative results on ergodic sets generated with the help of GDSC. The 49-vertex graph *X* on the left has *n*_*s*_ = 3 subgraphs *X*_*i*_, 1 ≤ *i* ≤ *n*_*s*_, shown in blue, orange, and maroon. There are three results implied by experiments on smaller graphs and theory-based extensions to larger graphs; see text for details. All three of these results significantly extend characterizations of ergodic sets.

We focus here on three general results that we proved from these experiments [9], which are stated in terms of the example in Fig 7 (for concreteness). There are *n*_*c*_ = 9 cliques in the graph on the left, and vertices can take on the states in *K* = {0,1}. There are *n*_*s*_ = 3 subgraphs *X*_*i*_, *i* ∈ {1,2,3}, in different colors, with *X*_1_, *X*_2_, and *X*_3_ containing *n*_*c*,1_ = 2, *n*_*c*,2_ = 4, and *n*_*c*,3_ = 3 cliques, respectively, that form the graph *X*. Our results follow.

1. There are $2_{c}^{n}=2^{9}$ fixed points (attractors) in the graph of Fig 7.
2. Let all vertices in *X* be in state 1, except those vertices with the gray background, which are in state 0. However, the vertices labeled 1, 2, and 3—called free boundary vertices (FBVs)—with the gray background can be in either state 0 or state 1. This produces ergodic sets that are binary hypercubes $Q_{2}^{n_{s}}$, with $2_{s}^{n}=8$ vertices. The hypercube on the right of Fig 7 denotes the state (*x*_1_, *x*_2_, *x*_3_) of FBVs 1, 2, and 3 (other vertex states are fixed, as just described, and not shown for clarity).
3. There are at least $n_{Q}=\prod_{i=1}^{n_{s}}n_{c,i}=2\cdot 4\cdot 3=24$ distinct hypercubes with this structure that vary with different choices of FBVs.

The key insight can be understood by considering the two *K*_6_ cliques on the left in the figure that comprise *X*_1_. If all vertices in the upper clique are in state 1 and all the vertices in the lower clique are in state 0, then we see that because the thresholds are (*k*_01_, *k*_10_) = (*q*_*i*_ − 1, *q*_*i*_ − 1) = (5,5), we achieve fixed points when vertex 1 is in state 0 and state 1. Thus, we can flip that state back and forth and remain in different fixed points. The construction of the ergodic sets then follows [9].

Theoretical results (2) and (3), which are presented more formally in [9], illustrate that GDSs can be specified to generate (*i*) an arbitrarily large number of ergodic sets, and (*ii*) these ergodic sets can have an arbitrarily large number of vertices; i.e., an arbitrarily large number of system states. Both results are significant departures from previous work, and suggest much greater levels of biodiversity and evolutionary potential. Furthermore, the cliques themselves are suggestive of communities or clusters of cells.

This particular example illustrates another point. The graph of Fig 7 contains 49 vertices. To produce these results, we run each subgraph *X*_*i*_ with GDSC independently. The dashed lines represent edges that serve to produce a connected graph. However, these edges are chosen so that there is no interaction among the *X*_*i*_ and hence they can be evaluated separately. The point is, larger graphs can be analyzed by exploiting problem semantics, as stated in the Introduction.

We make several points regarding the use of GDSC in these three examples. First, our experiments and theoretical results include synchronous, sequential, and block sequential update schemes, illustrating the utility of being able to model all of these update disciplines. Second, since these results were generalized through rigorous proofs, they apply to graphs of any finite size (e.g., *n* > 1 million vertices), which is well beyond the sizes of graphs that can be fully characterized experimentally. Third, these results are also applicable to system control issues [32].

### Biological Networks

**Fig 8:**
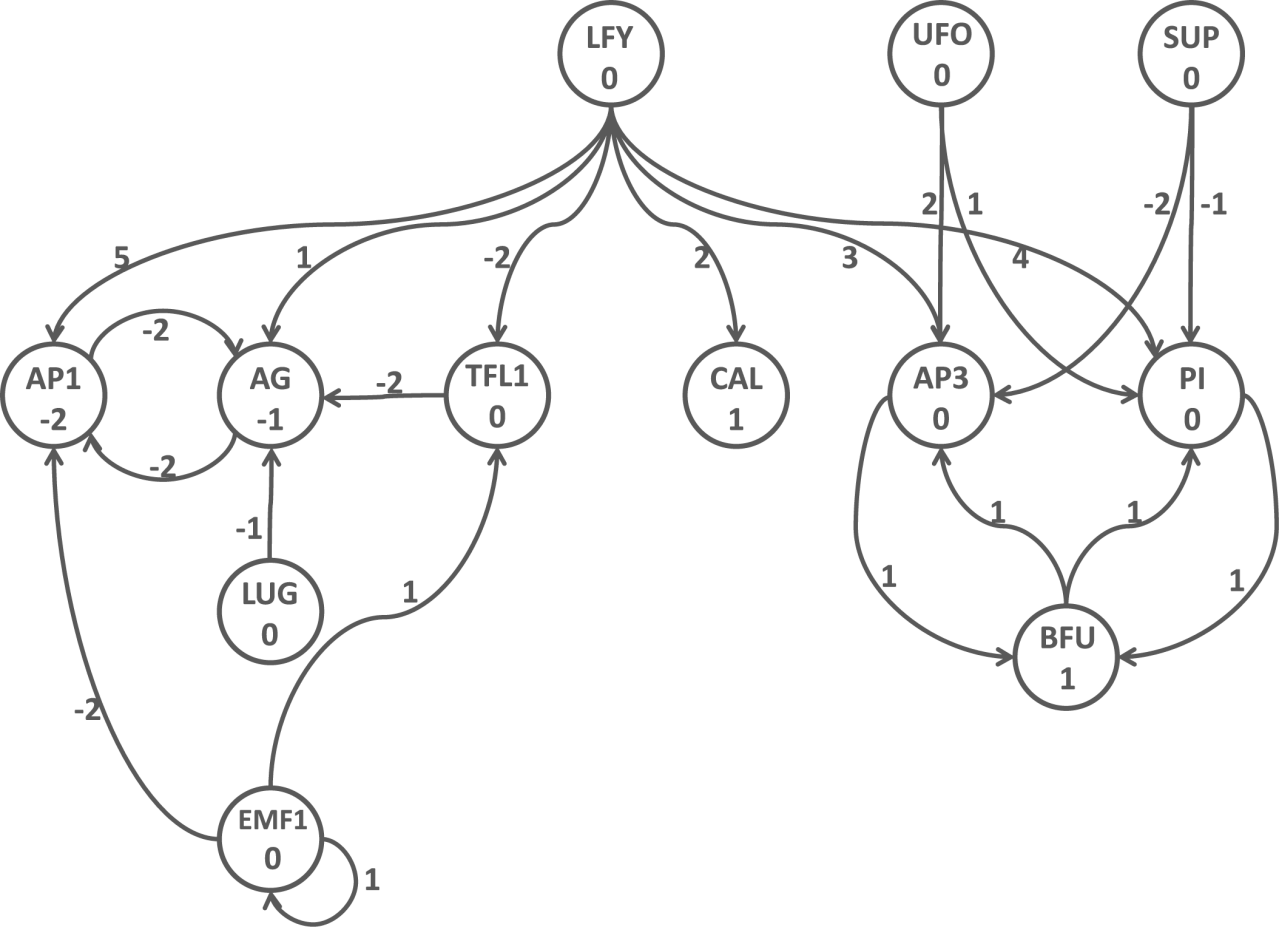
Biological network from [5] modeled by GDSC. Edge weights are given next to edges, and vertex thresholds are specified inside the vertices.

Here, we study threshold automata Boolean networks (with *K* = {0,1}) that are used to model genetic regulatory networks (e.g., [5, 25]). Both forms of vertex state transition, namely 0 → 1 and 1 → 0, are permitted. In particular, we model the 12-vertex, weighted, directed graph in Fig 3 of [5], shown here as Fig 8. The linear threshold model used for the state transition dynamics for vertex *v* is given by

$$\begin{array}{c} f_{v}\left(x\right)=\sum_{u\in n[v]}\left(w_{u,v}x_{u}\right)-k_{v} \end{array}$$ (4)

with the next state of *x*_*v*_ given by

$$\begin{array}{c} x_{v}=\{\begin{array}{cc} 1 & \;\text{if}\;f_{v}\left(x\right)>0\\ 0 & \;\text{if}\;f_{v}\left(x\right)\leq 0 \end{array} \end{array}$$ (5)

Here, *w*_*u*, *v*_ is the weight of the directed edge from *u* to *v*, and denotes the influence of vertex *u* on *v*, and *k*_*v*_ is the threshold of vertex *v*. The edge weights and vertex thresholds are given in Fig 8. A negative edge weight *w*_*u*, *v*_ means that *u* inhibits the transition of *v*.

In GDSC, we use the linear threshold vertex function with self-loops explicitly specified (only one vertex in Fig 8 has a self-loop). The limit sets (attractors) of [5] are six fixed points for the sequential case and six fixed points and seven 2-cycles for the synchronous case (see their Table 1). We reproduce their results: our sequential GDS produces 46 fixed points and our synchronous GDS produces 46 fixed points and 20 2-cycles; our limit cycles include all of their limit sets. The analyses with GDSC are provided in Table H in S1 File. They argue that their limit cycles are the only meaningful ones for their particular application. We can also model the other networks in that paper.

**Table 1:** Overview of selected dynamical systems software tools that are most closely aligned with GDSC.

Name	Vertex State Set Size	Complete Phase Space	Limit Cycles	Update Schemes	Number of Vertex Functions	Functional Equivalence	Cycle Equivalence	Type of System
GSDC	≥ 2	yes	yes	synchronous, sequential, block sequential	15 families, all deterministic	yes	yes	online collaborative environment
ADAM [52]	≥ 2	yes	yes	synchronous, sequential (for PDS only)	user-specified polynomials with logical operations; deterministic and stochastic	no	no	online individual user
FiatLux [40]	2	no	no	synchronous, sequential	12	no	no	individual user; Java app.
DDLab [43]	≥ 2	yes	yes	synchronous, sequential	many (tabular data)	yes, in CA	yes, in CA	individual user; C
BNS [41]	2	no	yes	synchronous	many	NA	NA	individual user
RBNLab [45]	≥ 2	yes	yes	various sequential schemes	many	no	no	individual user; Java app
Matlab RBN [44]	2	yes	yes	synchronous, asynchronous, sequential	many (tabular data)	no	no	individual user; Matlab
BoolNet R Package [49]	2	yes	yes	synchronous, sequential	many (tabular data)	no	no	run within R
GINsim [30, 53, 54]	2	yes	yes	synchronous, sequential	Boolean formulas with boolean operators	no	no	individual user; Java app
Chem-Chains [56]	2	yes	yes	synchronous, sequential	Boolean functions as truth table	no	no	individual user; C++
JCASim [62]	≥ 2	yes	yes	synchronous, sequential, block sequential	many	no	no	individual user; Java
GenYsis [60]	2	yes	yes	synchronous, sequential	many	no	no	individual user; C++
lnet [61]	2	yes	yes	sequential	Boolean functions as binary decision diagram	no	no	individual user; C

## Discussion

Features of GDSC are compared with those of some other discrete dynamical systems software. This overview is not exhaustive; an avenue for future work would be to conduct a thorough evaluation of all dynamical systems software. Our view is that different tools have different capabilities and are useful for particular types of problems. Hence, we will identify features of other tools that GDSC does not have. To do this, we first introduce some terminology.

### Sampling of Dynamical Systems

A **forward trajectory** is the sequence of state transitions, starting from a specified state, and continuing until a limit cycle is reached, or, in the case of probabilistic models, until some number of state transitions is completed.

There are multiple definitions for **asynchronous** update. We defined asynchronous update earlier. In [25, 33], asynchronous update is the process of selecting one vertex (at random) whose state is updated at each time *t*. A single vertex is typically selected uniformly at random, but other approaches are used, such as specifying *b* ≤ *n* vertices randomly at each time step to update. In [29, 34], a different definition is used: asynchronous update is characterized by each vertex having a different time interval between state updates.

**Cellular automata** (CA) are dynamical systems that use grid structures of cells, where each cell has a state and is influenced by its nearest neighbors (either 4—north, south, east, and west—or 8—where diagonal cells are included) [35]. A vertex in a dependency graph, which can have connections to any other vertices in the graph, is a generalization of the connectivity of a cell in a grid. As originally conceived, the cell state space was Boolean and the update scheme was synchronous. The local rule of a cell, analogous to a vertex function, computes the cell’s next state.

A **Boolean network** (BN), as originally conceived, is a dynamical system that is similar to a CA except that the regular grid is replaced by a graph where each vertex serves the role of a cell of a CA, and each vertex is connected to any number of the *n* graph vertices (including self-loops). Each vertex’s function is based on the number of edges incident on it, but as the name implies, the vertex state set is Boolean.

A **random Boolean network** (RBN) is a dynamical system that is random in two senses. First, the dependency graph (also called a **wiring diagram**) consists of directed edges (*u*, *v*) having the meaning that the state of vertex *u* is an input to the vertex function for *v*. Edges in the graph are specified randomly, but each vertex has the same in-degree *d* ^*in*^, and hence the same number of function arguments. Second, the vertex function of each vertex is assigned randomly. Functions are assigned once and remain fixed through computations of dynamics [19]. A common approach is to use elementary cellular automata (ECA) rules, where each function has three inputs [36]. There are many variants of this basic model [33]. Once the graph and vertex functions are assigned, the dynamics are subsequently deterministic.

A **probabilistic Boolean network** (PBN) is a dynamical system in which there are multiple vertex functions specified for each vertex, and at each time (in a forward trajectory) one function is selected for execution according to its associated probability [11]. The selected functions may be executed synchronously or sequentially.

A **polynomial dynamical system** (PDS) is a discrete dynamical system wherein each vertex function consists of polynomials in the vertex states (operations are typically addition, multiplication, and exponentiation) [37]. These types of systems, while some of the most common, are not the only types of dynamical systems; other dynamical systems can be defined to suit particular needs.

### Sampling of Dynamical Systems Tools

Table 1 summarizes selected properties of some of the tools discussed herein. We first describe some stand-alone (desktop) tools, focusing on CA, BNs, and RBNs. Mathematica [38] has CA capabilities. Tools such as Dynamica [39] have been built on top of Mathematica. FiatLux [40] is a CA simulator that sits on top of Ptolemy. It is used to study system robustness. It provides ten graph types and 12 dynamics models that are applied uniformly to vertices. Sequential and synchronous update schemes are available. BNS (Boolean Networks with Synchronous update) [41] uses update functions that are given as truth tables. However, this flexibility comes at a cost; the size of each truth table is exponential in the number of inputs to the function. It computes attractors (i.e., limit cycles), with a technique that does not compute all state transitions.

Another CA and RBN tool is DDLab [42, 43]. This tool can evaluate a variety of functions, including elementary cellular automata (ECA) rules, and includes sequential and synchronous update schemes. A discriminating and impactful feature of this stand-alone code is the plots that it generates. To the best of our knowledge, its visualizations are well beyond those of most other systems (including GDSC) and include state-time and Derrida plots, as well as 3-dimensional graphics. It has additional features, such as the ability to run some dynamical systems backwards; to determine predecessors of particular states; to compute functional and cycle equivalence on CA; and to compute attractor graphs. It directly computes phase space for networks with 30 or fewer vertices, and (statistically) computes limit cycles and transients for larger networks.

An RBN toolkit for Matlab that will handle synchronous and sequential update is available [44]. It uses tables to specify vertex functions. It also has significant visualization capabilities. Furthermore, it implements many of the variant RBN models described in [33], as well as others. Another RBN software is RBNLab [45]. It, too, implements a wide range of RBNs, well beyond the classic RBN described earlier [46]. GDSC, by comparison, implements CA and BNs. It can also execute some RBNs (e.g., where each vertex has in-degree three), but the specification of random edges and the assignment of random vertex functions must be done outside of GDSC.

There are other stand-alone software systems focused on PBNs. There are toolkits within Matlab [47] that are useful in analyzing particular dynamical systems; e.g., the Probabilistic Boolean Network toolkit developed by I. Shmulevich’s research team [48]. The BoolNet tool [49] is a package that works within the R statistical software. It evaluates synchronous and asynchronous Boolean networks and PBNs. Vertex functions are based on logical rules (using AND, OR, and NOT). Interestingly, it will also (re)construct (approximately) a graph that gives rise to a specified time series of state transitions. GDSC currently does not implement PBNs; we have a different software tool for stochastic systems.

All tools mentioned thus far are open-source software packages, or add-ons to commercial products, that run on a user’s machine, and in this sense are stand-alone applications.

We mention in passing that the Ptolemy project at UC-Berkeley has developed software to model large physical systems [50] used in signal processing, telecommunications, network design, investment management, and can analyze both continuous and discrete systems [51].

ADAM [52] is a web-based application. It is tailored for biological networks, and provides several of these, but can work with other types of networks. Among its models are Petri nets and PBNs. Its novelty, with respect to dynamical systems, is the use of PDSs, where vertex functions are polynomials in the vertex states. PDSs use both synchronous and sequential update. Functions are entered in symbolic format, so there is considerable flexibility in vertex functions (as long as they are polynomial in the inputs). For PDS, ADAM computes the entire phase space for graphs up to 20 vertices, and computes only limit cycles for larger graphs. However, the system supports about 100 state values for each node. Their BN permits synchronous update; the vertex functions are combinations of logical operators (AND, OR, NOT). The entire phase space is computed for BNs.

GINsim [30, 53, 54] is a Java application for simulating (regulatory) networks that use two types of graphs: logical regulatory graphs and state transition graphs for the analysis of logical models. The vertex state set can be of any size, based on the number of expression levels [30]. GINsim can perform asynchronous and synchronous updates with multivalued logical functions.

Cell Collective [55] is a web-based tool that simulates biochemical processes. That is, it computes forward trajectories of successive system states from a provided initial state. It promotes collaboration among scientists for building large scale biological models (e.g., models with thousands of nodes). There are several features, such as a Knowledge Base that includes a model repository for public use, and Bio-Logic Builder to build models, that enable researchers to explore different models and test hypotheses. The Cell Collective uses the ChemChains simulation engine [56].

We briefly mention several other tools. CellNetAnalyzer (CNA) [57] is a MATLAB toolbox for understanding structural and functional properties of metabolic, signaling, and regulatory networks. CNA is the extension of FluxAnalyzer [58] (originally developed for metabolic network and pathway analysis). MaBoSS [59] is a C++ tool that models biological networks using continuous time Markov processes applied on a Boolean state space. MaBoSS provides a high level language to describe Boolean equations. GenYsis [60] is another tool for analyzing the steady states of biological (gene regulatory) networks. Its vertex functions include logical operators. lnet [61] is another tool for Boolean networks that enumerates fixed points and limit cycles for up to 20 vertices; it performs approximate computations for networks of up to 70 vertices. JCASim [62] is a general-purpose Java simulator in Java. It supports different lattice structures (1-D, 2-D square, hexagonal, triangular, 3-D), neighborhoods, and boundary conditions, and can display the cells using colors, text, or icons. NetBuilder [63] offers capabilities to simulate and analyze regulatory networks. It represents networks using Petri nets and uses a genetic algorithm to evolve genetic regulatory networks (GRNs) needed for specific behavior. Pybool [64] is a Python-based tool for simulation. It helps biologists to define restrictions and conditions to limit the space of networks to ones that are most promising for further experimentation. Pybool uses the IPython package for parallelization. Golly [65] is a tool that simulates Conway’s Game of Life and many other types of cellular automata. Ready [66] is a tool that is used for continuous and discrete cellular automata. Ready supports 1D, 2D, 3D, polygonal and polyhedral meshes. Ready relies on OpenCL [67] as a computation engine.

GDSC is a web-based application and a modeling environment. That is, besides alleviating the need to compile code and maintain it, or purchasing third-party software such as Matlab, we store results on our clusters so users need not concern themselves with data storage, which can be a significant concern since a result file for one analysis can be gigabytes in size. Our post-processing capabilities for functional and cycle equivalences are somewhat unique, although software systems such as DDLab generate these plots for CA, as well as Derrida and other plots. We produce an XML output file of all results, which can be used by other software systems to generate Derrida and other results.

With respect to dynamical systems capabilities, we also have discriminating features. To our knowledge, GDSC is the only system that incorporates the block sequential update scheme and unfair word ordering (in addition to synchronous and sequential disciplines). Furthermore, vertex functions can be any functional form that can be coded in a high level programming language (i.e., C++). This means, for example, that we need not use functions that result in Markovian processes, where the next state of a vertex depends only on the current state of vertices. That is, we can introduce history dependence. However, functions must be coded by a GDSC team member and compiled into the code; users cannot currently specify vertex functions on-the-fly, which is another limitation. We provide 15 families of vertex functions from which a user chooses.

## Conclusions

An open access, distributed web-based application, GDSC, has been described in terms of its mathematical foundations, and illustrative research-driven examples have been presented to demonstrate its utility. Our current focus is phase space computations, since these results facilitate our mathematical work on dynamical systems. The system complements other dynamical systems software tools by providing features that other systems do not—and other tools have features that GDSC does not possess.

## Supporting Information

S1 File Supporting information: compilations of input and output files of analyses conducted with GDSC. (PDF)

S2 File Archive file containing GDSC input and output files for analyses using the nor vertex functions. (GZ)

S3 File Archive file containing GDSC input and output files for analyses using dependency graphs in the form of trees. (GZ)

S4 File Archive file containing GDSC input and output files for analyses where the vertex state set is *K* = {0,1,2}. (GZ)

S5 File Archive file containing GDSC input and output files for analyses using bithreshold vertex functions to compute a phase space and an attractor graph. (GZ)

S6 File Archive file containing GDSC input and output files for analyses used to determine ergodic sets. (GZ)

S7 File Archive file containing GDSC input and output files for analyses used to determine phase spaces of a biological network. (GZ)
