# Supplementary material for: GDSCalc: A Web-Based Application for Evaluating Discrete Graph Dynamical Systems
Source: PLoS One. 2015 Aug 11;10(8):e0133660. doi: 10.1371/journal.pone.0133660 (PMC4532456; doi:10.1371/journal.pone.0133660)
Supplement: S1 File — (PDF) [file pone.0133660.s001.pdf]

GDSCalc: A Web-Based Application for Evaluating Discrete Graph Dynamical Systems

Sherif H. Elmeligy Abdelhamid[1], Chris J. Kuhlman[2*], Madhav V. Marathe[2], Henning S. Mortveit[2], S. S. Ravi[3]
1 Computer Science Department, Virginia Tech, Blacksburg, Virginia, USA
2 Virginia Bioinformatics Institute, Virginia Tech, Blacksburg, Virginia, USA
3 Computer Science Department, University at Albany—SUNY, Albany, New York, USA
∗ E-mail: Corresponding ckuhlman@vbi.vt.edu (CJK)

Supporting Information

There are six archives (files) of analyses performed with GDSC that support the results in this paper. These are S2 File through S7 File, and are given in the table captions below. In each file are all input files for a set of analyses, and all output files for each analysis. The main input file and all output files for one GDSC analysis have the same name, for easy connection between inputs and outputs. For example, consider the third row in Table A. The base name in the Supporting Information (SI), for all of these files, is *nor.circ.4.blk.seq.01*. The particular files with this base name are: *nor.circ.4.blk.seq.01.inp* (the main input file), *nor.circ.4.blk.seq.01.xml* (the XML output file), *nor.circ.4.blk.seq.01.out* (a plain text output file that summarizes limit cycles), *nor.circ.4.blk.seq.01.timing.out* (timing data for analysis execution), and *nor.circ.4.blk.seq.01.gds.maxcyclelength.out* (integer representing the largest limit cycle). All of these files are in S2 File, per the caption of Table A. Each table below provides a set of analyses in one of the compressed supporting information files, cross-referenced by the Analysis ID within GDSC. All of these analyses in GDSC are public, and hence can be viewed by all users.

We note that in the software, we use zero-indexing for the vertices in a dependency graph, whereas in the theory, the convention is to use one-indexing. This means that a 4-vertex graph within GDSC will have vertices 0, 1, 2, and 3, while in the manuscript, we use vertices 1, 2, 3, and 4. One place where this difference comes into play is for permutations. Within GDSC we might specify the permutation $\pi = (3, 0, 2, 1)$, which would appear in the manuscript as $\pi = (4, 1, 3, 2)$.

Table A. Analyses for nor vertex functions. The archive file for these files is S2 File.

GDSC Analysis ID	Base Name in SI Files	Description
765	nor.circ.4.seq.01	Sequential analysis on Circle_4.
766	nor.circ.4.syn.01	Synchronous analysis on Circle_4.
767	nor.circ.4.blk.seq.01	Block sequential analysis on Circle_4.

Table B. Analyses for X-trees and sequential update. The archive file for these files is S3 File.

GDSC Analysis ID	Base Name in SI Files	Description
768	bit.bin.x.5.nodes.seq.01	X-tree analysis for graph with 5 vertices.
769	bit.bin.x.9.nodes.seq.01	X-tree analysis for graph with 9 vertices.
770	bit.bin.x.13.nodes.seq.01	X-tree analysis for graph with 13 vertices.
771	bit.bin.x.17.nodes.seq.01	X-tree analysis for graph with 17 vertices.

Table C. Analyses for Y-trees and sequential update. The archive file for these files is S3 File.

GDSC Analysis ID	Base Name in SI Files	Description
772	bit.bin.y.4.nodes.seq.01	X-tree analysis for graph with 4 vertices.
773	bit.bin.y.7.nodes.seq.01	X-tree analysis for graph with 7 vertices.
774	bit.bin.y.10.nodes.seq.01	X-tree analysis for graph with 10 vertices.
775	bit.bin.y.13.nodes.seq.01	X-tree analysis for graph with 13 vertices.
776	bit.bin.y.16.nodes.seq.01	X-tree analysis for graph with 16 vertices.

Table D. Analyses for H-trees and sequential update. The archive file for these files is S3 File.

GDSC Analysis ID	Base Name in SI Files	Description
777	bit.bin.h.6.nodes.seq.01	H-tree analysis for graph with 6 vertices.
778	bit.bin.h.10.nodes.seq.01	H-tree analysis for graph with 10 vertices.
779	bit.bin.h.14.nodes.seq.01	H-tree analysis for graph with 14 vertices.
780	bit.bin.h.18.nodes.seq.01	H-tree analysis for graph with 18 vertices.

Table E. Analyses for determining limit sets (attractors) for Circle graphs where vertices have states in $K = \{0, 1, 2\}$, with changes in k_{01}. The archive file for these files is S4 File.

GDSC Analysis ID	Base Name in SI Files	Description
810	3st.k01.2.circ.5.nodes.seq.01	Sequential update with $k_{01} = 2$ on Circle$_5$.
811	3st.k01.1.circ.5.nodes.seq.01	Sequential update with $k_{01} = 1$ on Circle$_5$.
812	3st.k01.2.circ.10.nodes.seq.01	Sequential update with $k_{01} = 2$ on Circle$_{10}$.
813	3st.k01.1.circ.10.nodes.seq.01	Sequential update with $k_{01} = 1$ on Circle$_{10}$.

Table F. Analyses for determining limit sets (attractors) and the attractor graph for the Circle$_4$ graph, with bithreshold vertex functions. The archive file for these files is S5 File.

GDSC Analysis ID	Base Name in SI Files	Description
782	bit.bin.1.3.circ.4.seq.01	Sequential update with $(k_{01}, k_{10}) = (1, 3)$ on Circle$_4$.

Table G. Analyses for determining ergodic sets using a bithreshold model with synchronous update. These three analyses combine to provide results for the overall system. The archive file for these files is S6 File.

GDSC Analysis ID	Base Name in SI Files	Description
784	bit.bin.5.5.2.k6.syn.01	Subgraph with two K_6 cliques.
785	bit.bin.4.4.4.k5.syn.01	Subgraph with four K_5 cliques.
786	bit.bin.x.y.2.k6.1.k5.syn.01	Subgraph with two K_6 and one K_5 cliques.

Table H. Analyses for determining limit sets (attractors) for a biological network using the linear threshold model. The archive file for these files is S7 File.

GDSC Analysis ID	Base Name in SI Files	Description
804	biology.lt.12.nodes.syn.01	Synchronous update.
805	biology.lt.12.nodes.seq.01	Sequential update.
